# Comparing German medical guidelines with recommendations by large language models using the example of hemolytic uremic syndrome (TMA)

**DOI:** 10.1007/s00467-025-07008-5

**Published:** 2025-10-18

**Authors:** Adrian Gieser, Samipa Pudasaini, Dominik Müller

**Affiliations:** Department of Pediatric Gastroenterology, Nephrology and Metabolic Disorders, Campus Virchow-Klinikum and Campus Charité Mitte, Charité - Universitätsmedizin Berlin, Corporate Member of Freie Universität and Humboldt-Universität zu Berlin, Berlin, Germany

With the rapid advancement of large language models (LLMs), such as the Generative Pre-trained Transformer (ChatGPT), a new form of artificial intelligence has entered both society and medicine. Due to their ability to generate arbitrary text on demand, answer scientific questions, and summarize complex content, LLMs have become increasingly relevant in healthcare. Medical professionals often use LLMs to obtain information regarding diagnostics and treatment, as data retrieval via LLMs is often faster than via conventional guideline-based research. This is particularly relevant in rare diseases within highly specialized fields such as pediatric nephrology, where comprehensive resources are less accessible. Recently, Nadide Sav published an article on this topic, concluding that GPT LLMs (versions 3.5 and 4) did not outperform practicing pediatric nephrologists when responding to clinical questions [1]. Building on this, the extent to which LLM-generated recommendations align with established medical guidelines in pediatric nephrology has not yet been systematically assessed. Existing studies from other specialties (e.g., lumbosacral radicular pain, atopic dermatitis, lower back pain) have shown moderate-to-low concordance between LLM outputs and clinical guidelines [2–4].

This research gap is addressed in the present study. We evaluated the consistency of LLM-generated answers to clinical questions with recommendations from current medical guidelines and examined potential differences in response quality between different LLMs. ChatGPT (version 4o) and Perplexity (version Standard Web 01.2025) were selected as representative models due to their widespread use. A total of 24 questions were derived from 12 recommendations of the national S2k guideline of the *Association of the Scientific Medical Societies in Germany* (AWMF) for hemolytic uremic syndrome (HUS), recently subsumed under the broader term thrombotic microangiopathy (TMA). This guideline was issued by the *German Pediatric Nephrology Association* (GPN) [5]. Additionally, 10 questions based on 5 real patient cases from our department were submitted to both LLMs. Answer quality was evaluated based on concordance with guideline recommendations using a 5-point Likert scale. Statistical analysis was performed using the Kolmogorov–Smirnov test and the Mann–Whitney *U* test via the SPSS software (version 30). A *p*-value < 0.05 was considered statistically significant. A diagram of the study design and results is provided in Fig. 1.

**Fig. 1:**
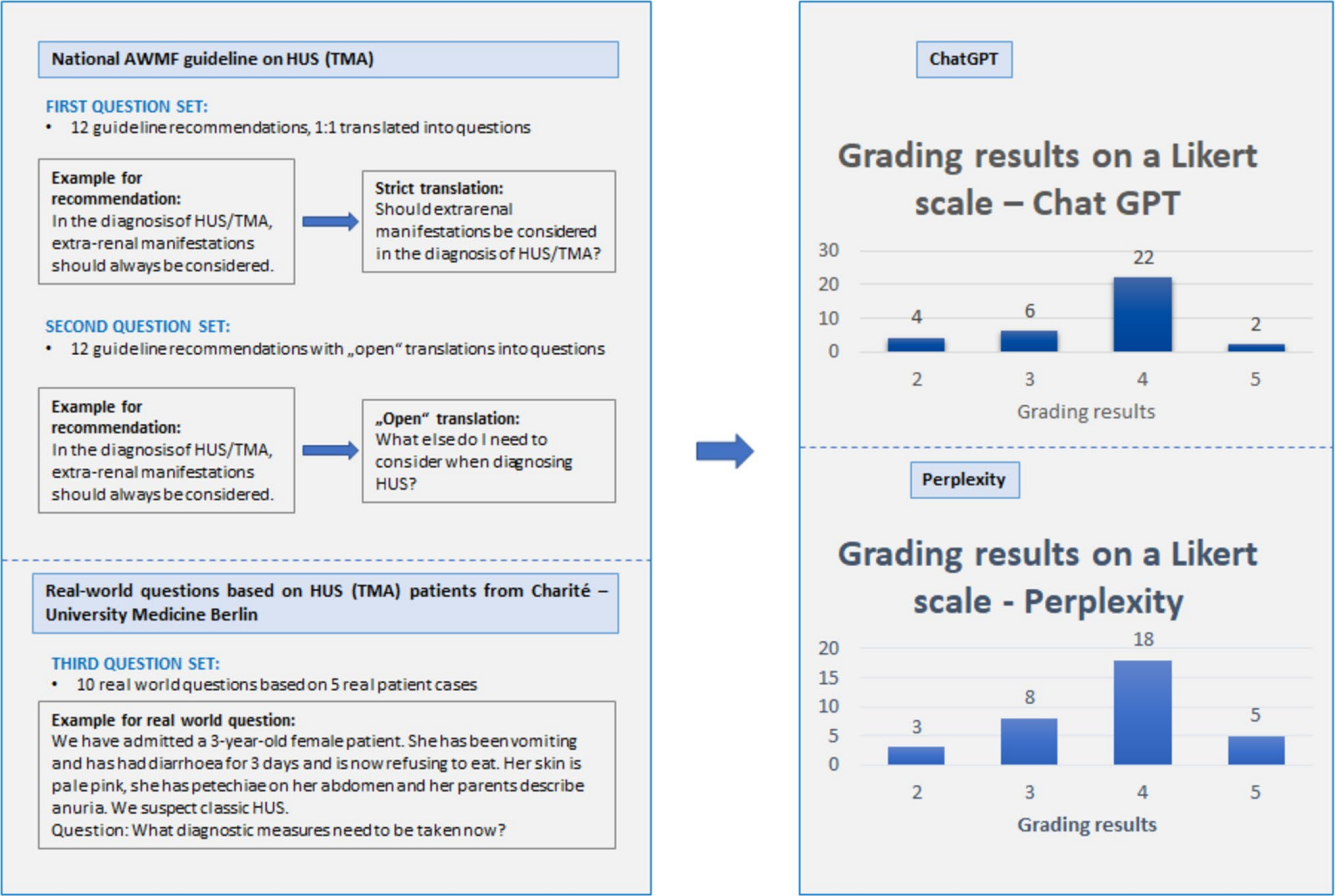
Study methodology and grading results. *HUS* hemolytic uremic syndrome, *TMA* thrombotic microangiopathy.

No statistically significant difference in response quality was observed between ChatGPT and Perplexity (*p* = 0.728). Both models produced answers with reasonable accuracy but were not fully consistent with guideline recommendations. In total, 67.65% of Perplexity’s responses and 70.59% of ChatGPT’s responses were in full or near-verbatim agreement (grade 4 or 5) with the guideline content. While Perplexity cited references appropriately, it did not differentiate clearly between sources.

This study focused on the rare pediatric nephrological condition HUS (TMA). Our findings suggest that the current error rate of LLMs is still too high to recommend their standalone use as clinical decision-support tools. Guidelines continue to represent the most reliable information source, mainly due to their transparent citation of evidence. A potential middle ground may be the approach proposed by Tariq et al., in which LLMs were fine-tuned by being trained with clinical guideline content, resulting in a reported accuracy of 100% [6].

## Data Availability

Data will be made available upon reasonable request.

## Abbreviations

*HUS*: hemolytic-uremic syndrome
*TMA*: thrombotic microangiopathy
